# Marine Polysaccharides: A Source of Bioactive Molecules for Cell Therapy and Tissue Engineering

**DOI:** 10.3390/md9091664

**Published:** 2011-09-23

**Authors:** Karim Senni, Jessica Pereira, Farida Gueniche, Christine Delbarre-Ladrat, Corinne Sinquin, Jacqueline Ratiskol, Gaston Godeau, Anne-Marie Fischer, Dominique Helley, Sylvia Colliec-Jouault

**Affiliations:** 1 Seadev-FermenSys SAS, Technopole Brest Iroise, 185 rue René Descartes, Plouzané 29280, France; E-Mail: senni@seadev.fr; 2 INSERM U765, Faculté de Pharmacie, UMR-S765, Université Paris Descartes, Sorbonne Paris Cité, 4 avenue de l’Observatoire, Paris 75006, France; E-Mails: jessica.pereira@etu.parisdescartes.fr (J.P.); anne-marie.fischer@egp.aphp.fr (A.-M.F.); dominique.helley@egp.aphp.fr (D.H.); 3 Biochemistry Department, Faculty of Dental Surgery, Paris Descartes University, Montrouge 92120, France; E-Mails: gueniche_farida@yahoo.fr (F.G.); godeau_g@yahoo.fr (G.G.); 4 Laboratory of Biotechnology and Marine Molecules, Ifremer, Rue de l’Ile d’Yeu, BP 21105, Nantes Cedex 03 44311, France; E-Mails: Christine.Delbarre.Ladrat@ifremer.fr (C.D.-L.); Corinne.Sinquin@ifremer.fr (C.S.); Jacqueline.Ratiskol@ifremer.fr (J.R.); 5 AP-HP, Biological Hematology Department, European Hospital Georges Pompidou, 20 rue Leblanc, Paris 75015, France

**Keywords:** marine bacteria, marine algae, exopolysaccharides, sulfated polysaccharides, structure, chemical modification, biological activity, blue biotechnology, cell therapy, tissue engineering

## Abstract

The therapeutic potential of natural bioactive compounds such as polysaccharides, especially glycosaminoglycans, is now well documented, and this activity combined with natural biodiversity will allow the development of a new generation of therapeutics. Advances in our understanding of the biosynthesis, structure and function of complex glycans from mammalian origin have shown the crucial role of this class of molecules to modulate disease processes and the importance of a deeper knowledge of structure-activity relationships. Marine environment offers a tremendous biodiversity and original polysaccharides have been discovered presenting a great chemical diversity that is largely species specific. The study of the biological properties of the polysaccharides from marine eukaryotes and marine prokaryotes revealed that the polysaccharides from the marine environment could provide a valid alternative to traditional polysaccharides such as glycosaminoglycans. Marine polysaccharides present a real potential for natural product drug discovery and for the delivery of new marine derived products for therapeutic applications.

## 1. Introduction

Sulfated polysaccharides have diverse functions in the tissues from which they originate. They are capable of binding with proteins at several levels of specificity and are involved mainly in the development, cell differentiation, cell adhesion, cell signaling and cell matrix interactions. These bioactive molecules present a great potential for medical, pharmaceutical and biotechnological applications such as wound dressings, biomaterials, tissue regeneration and 3D culture scaffolds and even drugs. The most studied for their biological properties are mammalian sulfated polysaccharides or glycoconjugates constituted by glycosaminoglycans (GAGs) composed of negatively charged osidic chains, most of them covalently linked to proteins. The discovery of the biological importance of the mammalian glycoconjugates has been the beginning of a new modern research field focusing on the carbohydrate based recognition phenomena, glycobiology [1,2]. It has been demonstrated that these particular biological properties were due to the chemical diversity of the osidic chains in which the patterns of sulfate substitution can give specific biological functions. It was also noted that the chemical diversity of the sulfated polysaccharides was largely species specific [3].

Marine polysaccharides present an enormous variety of structures; they are still under-exploited and they should therefore be considered as an extraordinary source of chemical diversity for drug discovery [4]. Sulfated polysaccharides, possessing GAG-like biological properties, can be found either in marine eukaryotes or in marine prokaryotes. This marine origin should offer potentially safer compounds than mammalian polysaccharides for drug discovery. In this review, we will present first the biological properties of GAGs from mammalian origin in relation to cell therapy and tissue engineering. Then we will describe different studies made on marine polysaccharides, showing that these polysaccharides could advantageously replace mammalian GAGs in some therapeutic applications.

## 2. Glycosaminoglycans: Structural Features, Biological Properties and Limitations for Therapeutic Use

Glycosaminoglycans (GAGs) are present in all animals; some of them such as heparin and dermatan-sulfate are extracted from mammalian mucosa for therapeutical uses. GAGs can be located in the extracellular matrix, on the cell surface or within the intracellular compartment. These polysaccharides are composed of disaccharide repeating units including one uronic acid (or neutral sugar for one of them) and one amino sugar. GAGs can be sulfated (chondroitin-sulfate, dermatan-sulfate, heparin/heparan-sulfate, keratan-sulfate) or not (hyaluronic acid). Furthermore, sulfated glycosaminoglycans can be covalently bound to a protein to form proteoglycans. Hyaluronic acid (HA) is very peculiar because it is neither sulfated, nor covalently linked to a protein to form proteoglycan. Moreover this polysaccharide has a very high molecular weight (up to 8 × 10^6^ g/mol in tissue) in contrast to sulfated GAGs (from 10 to 100 × 10^3^ g/mol) [5,6]. GAGs interact with a wide range of proteins involved in physiological and pathological processes. They display many biological activities which can influence tissue repair as well as inflammatory response [1,7–9].

**Figure 1:**
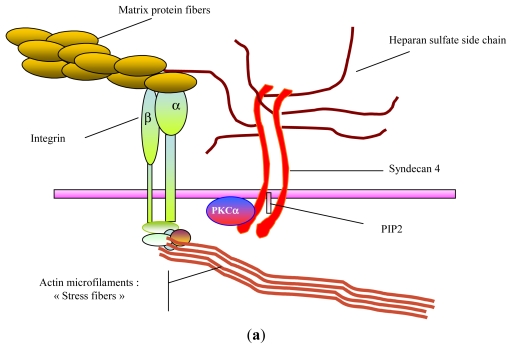

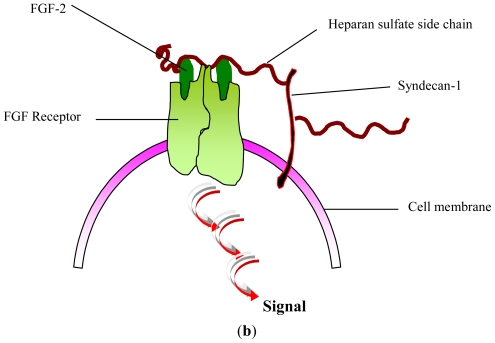
Cell surface GAGs and cell behavior. (**a**) GAGs and cell adhesion; (**b**) GAGs and growth factor promotion.

For example, heparan-sulfate chains borne by the cell surface proteoglycans are required to mediate signals of heparin-binding growth factors such as fibroblast growth factors (FGFs), heparin binding-epidermal growth factor (HB-EGF) or vascular endothelial growth factor (VEGF) (Figure 1) [10–12]. Furthermore in extracellular matrix, sulfated glycosaminoglycans are specifically involved in growth factor bioavailability and protection against proteinase degradation [13,14]. Their ability to structure matrix macromolecules has been described on collagens as well as on matrix glycoproteins or elastic fibers [15–17]. Other studies demonstrated that heparin and chondroitin-sulfate directly inhibit serine-proteinase activity and modulate matrix metalloproteinase activity in cell culture [18,19]. Due to specific interactions with chemokines and selectins, heparan-sulfates found on endothelial cell surface are also major actors in leukocyte rolling and chemo-attraction [20,21].

The HA, a non-sulfated GAG, is also a major actor in tissue structuring and remodeling. The hallmark of this polysaccharide is its ability to form hydrogels allowing joint lubrication and space creation to cell migration during wound healing and embryonic morphogenesis [22]. During cutaneous wound healing, HA prevents fibrosis [23]. Thus, during fetal skin repair, which is characterized by scarless wound healing, high amounts of HA are produced whereas a dramatic downregulation of this GAG synthesis is observed in the keloids [24]. HA stimulates endothelial cell proliferation, migration and differentiation following activation of specific cell receptors (CD44 receptor and hyaluronan-mediated motility receptor or RHAMM). HA is also involved in inflammatory response after injury by stimulation of macrophage cytokine secretion through CD44 signaling pathway [21].

Thus, these polysaccharides could be advantageously proposed as pharmacological agents or biomaterials for tissue repair and engineering. Unfortunately, the strong anti-coagulant activity of some of them (heparin, heparan-sulfate), their animal origin increasing the risk for the presence of infectious agents such as viruses or prions, and an unreliable availability (cost, volume) restrict their use in Human. Marine organisms such as macroalgae, microalgae, bacteria, cyanobacteria, invertebrates and chordata offer a rich source of carbohydrates with original structures largely species specific.

## 3. GAG-Like Polysaccharides from Marine Eukaryotes

### 3.1. GAGs

Hyaluronic acid, chondroitin sulfate, dermatan sulfate and heparan sulfate can be found in marine invertebrates; they have been isolated from marine mollusks or echinoderms such as sea urchins or sea cucumbers (ascidians). GAGs can be extracted from marine mollusk such as *Amussium pleuronectus* (Linne). The structural characterization showed that they are sulfated like heparin and contain equivalent amount of uronic acid and hexosamine. They could be an alternative source of heparin [25]. The dermatan sulfates isolated from sea urchin and chondroitin sulfates from ascidians have the same backbone structures as the mammalian GAGs but possess different sulfation patterns [26,27]. In animal models, the fucosylated chondroitin sulfate obtained from sea cucumber was a promising molecule with possible beneficial effects in pathological conditions such as thrombosis and ischemia [27]. Chondroitin/dermatan sulfate hybrid chains extracted from shark skin showed a high affinity for growth factors and neurotrophic factors [28].

### 3.2. Alginate

Marine alginate is found in all brown seaweeds (Phaeophyceae) in a proportion of 18 to 40% of the total plant. Alginate is both a biopolymer and a polyelectrolyte and is considered to be biocompatible, non-toxic, non-immmunogenic and biodegradable. Alginate is a high-molecular weight (in the range 200–500 × 10^3^ g/mol) polyuronic acid composed of two types of uronic acid distributed as blocks of guluronic acid (GulA or “G”) or mannuronic acid (ManA or “M”) as well as heteropolymeric mixed sequences (GulA-ManA, usually alternating). Often commercial alginate is characterized by its “M:G” ratio. The alginate is known to form a physical gel by hydrogen bonding at low pH (acid gel) and by ionic interactions with divalent or trivalent ions, which act as crosslinkers between adjacent polymer chains. The alginate and alginate with chemical modifications on carboxyl or hydroxyl groups, present real promise for obtaining new biomaterials useful in cell immobilization, controlled drug delivery and tissue engineering [29,30]. Tailored alginate hydrogels have been studied to transplant cells such as chondrocytes and osteoblasts and improve neo-cartilage or neo-bone formation. The beneficial use of these modified alginate gels as biomaterials has been demonstrated in a number of *in vitro* and *in vivo* studies [31].

### 3.3. Fucoidans

#### 3.3.1. From Marine Echinoderms

Biological properties of sulfated fucoidans (or fucans) extracted from marine invertebrates such as sea urchins or sea cucumbers have been extensively studied. These polymers of l-fucose are homogeneous and unbranched and bear no substituent other than sulfate. As described for mammalian GAGs, they present anticoagulant and antithrombotic activities. They can act as a ligand for either L- or P-selectins like heparin or heparan sulfate. They also are active on cell growth, migration and adhesion [3].

#### 3.3.2. From Seaweeds

**Figure 2:**
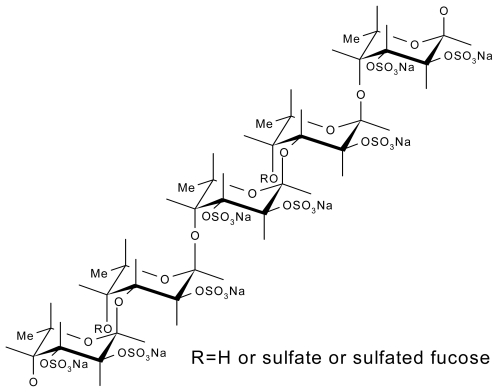
Structure of sulfated oligofucoidan constitutive of algal fucoidan from *Ascophyllum nodosum* [32].

Fucoidans can also be isolated from Phaeophyceae cell wall; algal sulfated fucoidans are more complex than fucoidans found in marine invertebrates. Algal fucoidans are composed of fucosyl disaccharide repeating units substituted by sulfates or uronic acids; they present other substituents such as *O*-acetyl, and branches adding considerably to their heterogeneity (Figure 2) [32–34].

**Figure 3:**
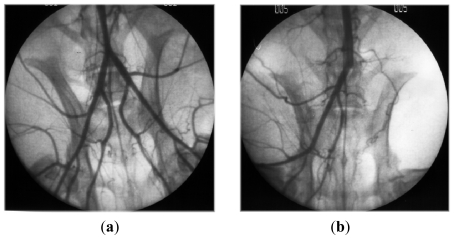
Angiographies of hind limbs from rabbits, 3 days after apoptosis induction. (**a**) Rabbit receiving LMWF; (**b**) Rabbit receiving placebo.

After depolymerization (by acidic hydrolysis or free radical process), low-molecular-weight fractions of fucoidans (LMW fucans, *<*30 kDa) have been obtained and shown to exhibit some heparin-like properties, with less side effects. Heparin is a sulfated polysaccharide from porcine origin used as an antithrombotic drug; however, its antithrombotic efficacy is limited by its strong anticoagulant properties correlated with a high hemorrhagic risk. The venous antithrombotic activity of LMW fucans (LMWF) has been compared with a low-molecular-weight heparin in the Wessler rabbit model and exhibited a better ratio antithrombotic effect/hemorrhagic risk [35–37]. Moreover LMWF exhibited arterial antithrombotic activity *in vivo* as well. Indeed, LMWF injections improved residual muscle blood flow and increased vessel formation in acute hind limb ischemia model in rat; they prevented arterial thrombosis induced by apoptosis in rabbit with no increase of bleeding risk (Figure 3) [38,39]. This antithrombotic activity may, in part, be explained by the decrease of tissue factor expression in the media of denuded arteries and the significant increase of plasma TFPI (inhibitor of the extrinsic coagulation pathway) released from endothelial cell by fucoidan as previously shown *in vitro* [39,40].

These results led us to further study sulfated polysaccharide-endothelial cell interaction. Owing to their ionic structure, LMWF, like heparin, can bind and modulate the activity of proangiogenic growth factors such as fibroblast growth factors (FGF) [41,42]. Fucoidan enhanced *in vitro* tube formation by mature endothelial cells in the presence of FGF-2 [43]. This effect correlated with a decrease of PAI-1 (plasminogen activator inhibitor) release, and an upregulation of the cell-surface α6 integrin subunit, which could explain the proangiogenic activity [42–44].

Until recently, vessel formation was claimed to be related to *in situ* mature endothelial cell proliferation, migration and differentiation. In 1997, Asahara and Isner demonstrated the presence of endothelial progenitor cells (EPCs) in circulating blood, which play a major role in vasculogenesis in physiological and pathological situations when organ vascularization, and regeneration, is required [45]. Autologous infusion of EPCs would potentially be a promising therapy for revascularizing ischemic tissues. Unfortunately these EPCs are very rare in blood. Moreover further evidence indicates that not only the cell number but also functional properties of transplanted EPCs determine the outcome of autologous stem cell transplantation. The poor graft efficiency seems to be related to unfavorable functional changes during the expansion procedure. We demonstrated that fucoidan induced EPCs to adopt a proangiogenic phenotype. It enhanced their proliferation, migration and differentiation into capillary-like structures on Matrigel [46]. LMWF could act through SDF-1, which when stimulated during EPCs expansion increased their therapeutic potential after cell transplantation in a model of hind limb ischemia [47,48].

LMWF has demonstrated some anti-inflammatory properties such as anti-complementary activities with both inhibition of leukocyte margination and connective tissue proteolysis. LMWF could be used for treating some inflammatory diseases in which uncontrolled extracellular matrix degradation takes place [49]. As described above, LMWF can also promote tissue rebuilding parameters such as signaling by heparin-binding growth factors (FGF-2, VEGF) and collagen processing in fibroblasts, smooth muscle cells or endothelial cells in culture. Recently, a study showed that LMWF can bind fibrillar collagens and provide protection and signal promotion of heparin binding growth factors to improve biocompatibility of purified cancellous bone substitute. Indeed, it was demonstrated that LMWF mimicks and restores the properties of bone non collagenous matrix (proteoglycans, glycoproteins) that were eliminated by drastic purification process during design of the biomaterial, to regulate soluble factors bioavailability [50].

## 4. GAG-Like Polysaccharides from Marine Prokaryotes

### 4.1. Extracellular Polymeric Substances (EPS)-Producing Cyanobacteria

Cyanobacteria (blue-green algae) are Gram-negative photosynthetic prokaryotes considered as a rich source of novel molecules of a great importance from a biotechnological and industrial point of view. Many cyanobacteria produce extracellular polymeric substances (EPS) mainly of polysaccharidic nature. These EPS can remain associated to the cell surface as sheaths, capsules and/or slimes, or be liberated into the surrounding environment as released polysaccharides [51].

### 4.2. Spirulina (Arthrospira)

*Spirulina* is a microalga which offers a broad range of applications such as a nutritive or pharmaceutical additive with no risk to health. Clinical studies suggest that compounds in the microalgae have therapeutic functions and especially polysaccharides with antiflammatory effects [52]. Spirulan, existing as a ionic form (calcium or sodium), is a sulfated polysaccharide isolated from *Arthrospira platensis* (formely *Spirulina platensis*) and consisting of two types of disaccharide repeating units, [→3)-α-l-Rha(1→2)-α-l-Aco-(1→] where Aco (acofriose) is 3-*O*-methyl-Rha with sulfate groups and *O*-hexuronosyl-rhamnose. It also contains trace amounts of xylose, glucuronic acid and galacturonic acid. Its molecular weight is about 200 × 10^3^ g/mol and it bears from 5 to 20% sulfate depending on the source [53]. Spirulan is reported to inhibit pulmonary metastasis in humans and to prevent the adhesion and proliferation of tumor cells. Highly porous scaffolds have been constructed by electrospining biomass of *Spirulina*. In these conditions, well defined nanofibers were produced to be used as extracellular matrices for stem cell culture and future treatment of spinal cord injury [54].

#### Other EPS-Producing Cyanobacteria

Cyanobacteria of the genera *Aphanocapsa*, *Cyanothece*, *Gloeothece*, *Synechocystis*, *Phormidium*, *Anabaena* and *Nostoc* are able to produce sulfated polysaccharides containing uronic acids. Applications of cyanobacterial polysaccharides have been poorly investigated in the biomedical field except as antiviral agents. Moreover, the overall negative charge of cyanobacterial EPS may be essential for sequestering metal cations that are essential for cell growth but present at low concentrations in their surroundings, and/or preventing the direct contact between the cells and toxic heavy metals dispersed in the environment [55].

### 4.3. Bacterial Exopolysaccharides (EPS)

Marine biosphere offers wealthy flora and fauna living in different unusual ecosystems embracing diverse microbial communities: deep-sea hydrothermal vents, Arctic and Antartic sea-ice, Mediterranean shallow vents, microbial mats located in some polynesian atolls [56–63].

Deep-sea microorganisms, bacteria and archaea, or the processes they mediate *in situ*, and the promise of their primary and secondary products present a great interest to biotechnology and a potential for pharmaceutical applications [64–66]. From different oceanographic cruises organised to explore deep-sea hydrothermal vent environments (East Pacific Rise, North Fiji, Guaymas basin and Mid Atlantic Ridge), several polysaccharide-producing bacteria have been discovered. Screenings have been performed mainly on mesophilic bacteria rather than on psychrophilic, thermophilic or hyperthermophilic strains. Up to date, three main genera of polysaccharide-producing bacteria have been identified: *Pseudoalteromonas* sp., *Alteromonas* sp. and *Vibrio* sp. By fermentation, each bacterium can liberate into the culture medium (an aerobic carbohydrate-based medium) one specific polysaccharide with an original structure and with an interesting yield, from 0.5 g to 4 g of polysaccharide/liter of culture broth [59].

#### 4.3.1. HE 800 EPS from *Vibrio diabolicus*

**Figure 4:**
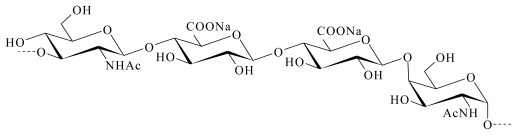
Repeating unit of the marine bacterial polysaccharide (HE800 EPS) produced by *Vibrio diabolicus* [68].

The *Vibrio diabolicus* bacterium was isolated from a Pompei worm tube (polychaete *Alvinella pompejana*); the EPS it secreted was characterized by equal amounts of glucuronic acid and hexosamine (*N*-acetyl glucosamine and *N*-acetyl galactosamine). It is a hyaluronic acid-like polymer (Figure 4) [67,68] and its commercial name is Hyalurift^®^.

The efficiency of this high-molecular-weight (>10^6^ g/mol) linear polysaccharide was evaluated on the restoration of bone integrity for critical size defects (CSD) performed on the calvaria of Wistar male rats. Collagen was used as a control. Briefly, bacterially produced polysaccharide or collagen (as control) was put into a hole made in the right parietal bone while another hole made in the left parietal bone was kept free of any compound. In the presence of EPS secreted by *Vibrio diabolicus*, bone healing was almost complete after 15 days; the anatomy of the defect with trabecular and cortical structure was totally restored. Neovascularization was also observed along with an organized trabecular bone. No abnormal bone growth or conjunctival abnormalities were noticed. Conversely, the collagen-treated animals did not demonstrate significant healing [69].

These results could be explained by the HE800 EPS effects observed subsequently in *in vitro* models of tissue remodeling. Indeed, it was demonstrated that HE800 EPS enhanced collagen structuring in engineering connective tissue model and promoted fibroblast settled in extracellular matrix. Using the capability of acido-soluble collagen I to auto-associate into fibrils after pH neutralizing, a reconstructed extracellular matrix containing human fibroblasts was produced and studied by electron microscopy and classical histology. By electron microscopy, it was observed that addition of HE800 EPS, during collagenous matrix building, increased and accelerated collagen fibrils formation with 67 nm periodic striations.

Study on fibroblasts distribution in different parts of reconstructed connective tissues demonstrated the ability of HE800 EPS to modulate signals mediated by cell-matrix interactions. In this *in vitro* model, proliferating cells were preferentially located at the tissue surface. In order to distinguish between fibroblasts colonizing the extracellular matrix and fibroblasts living and proliferating at the surface of the reconstructed tissue, stained histological sections were studied by image analysis. Observations showed that in reconstructed tissue containing HE800 EPS, cells at the periphery proliferated and massively migrated in the extracellular matrix. We could conclude that this EPS specifically improved cytocompatibility of the engineered tissue. Therefore, the use of HE800 EPS to design collagenic engineered tissue for skin or cartilage grafting can be suggested [70]. These efforts to design innovative medical devices or tissue engineering products demonstrate that HE800 EPS in native form could find application in the future as a new biomaterial for tissue therapy.

With the purpose of preparing a GAG-like compound, this high-molecular-weight EPS was first depolymerized by a free-radical reaction. Then the newly depolymerized EPS was chemically sulfated with pyridine-sulfur trioxyde in dimethylformamide. These chemical modifications can yield a low molecular weight (<20 kDa) and sulfated polysaccharide with new properties. In fact, depolymerization, *N*-deacetylation and sulfation produced a HE800 EPS derivative, referred to as DRS HE800, which is structurally close to heparan-sulfate. It was also demonstrated that other EPS having no GAG structural features could be modified in order to acquire heparan sulfate properties. The effect of this HE800 EPS derivative was tested in proliferation assays. Thus, in two-dimensional culture this derivative was capable of stimulating the proliferation of dermal and gingival fibroblasts. And moreover, this derivative could inhibit the secretion of matrix metalloproteinases (MMPs) such as gelatinase A (MMP-2) and stromelysin 1 (MMP-3) by fibroblasts after IL-1β induction [71].

**Figure 5:**
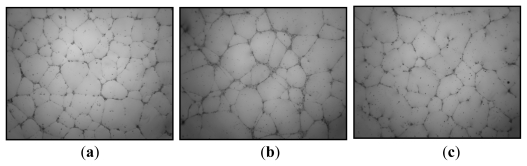
Effect of the DRS HE800 derivative on vascular tube formation on Matrigel from endothelial progenitor cells (EPCs). Photographs show vascular tube formation by EPCs previously treated (**a**) with 5% of fetal calf serum (control); (**b**) with proangiogenic factor VEGF (40 ng/mL); and (**c**) with proangiogenic factor VEGF (40 ng/mL) and DRS HE800 derivative (10 μg/mL).

Vascularization in tissue engineering is a challenge; indeed tissue repair is possible only in the presence of new vessels contributing to tissue growth. New vessels, contributing to vascular and tissue repair, are formed by two different processes. The first involves proliferation and migration of *in situ* mature endothelial cells. In the second process, the new vessels of proliferating, migrating, and differentiating endothelial progenitor cells (EPCs) from the bone marrow, are incorporated under the influence of proangiogenic factors such as VEGF. *In vitro* studies showed that DRS HE800 exhibited no proangiogenic properties (proliferation, migration, differentiation tested by vascular tube formation on Matrigel) either on mature endothelial cells such as HUVEC (human umbilical vein endothelial cells), or on EPCs [72] (Figure 5).

#### 4.3.2. EPS GY 785 from *Alteromonas infernus*

**Figure 6:**
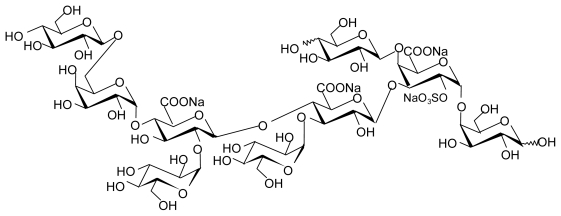
Repeating unit of marine bacterial polysaccharide (GY785 EPS) produced by *Alteromonas infernus* [74,75].

A bacterium named *Alteromonas infernus* was isolated from a sample of fluid collected among a dense population of *Riftia pachyptila*. The EPS it secretes is a highly branched acidic heteropolysaccharide with a high molecular weight (>1.5 × 10^6^ g/mol) and a low sulfate content (≤10%). Its nonasaccharide repeating unit is composed of uronic acid (galacturonic and glucuronic acid) and neutral sugars (galactose and glucose) and substituted with one sulfate group (Figure 6) [73,74].

A very recent study demonstrated that the high molecular weight GY785 EPS associated with an injectable silylated hydroxypropylmethylcellulose-based hydrogel (Si-HPMC) could significantly improve the mechanical properties of this new hydrogel construct. The attachment of chondrocytes and osteoblasts was induced when the cells were cultivated in two-dimensional culture on the top of the hydrogel containing 0.67% of GY785 EPS. In three-dimensional culture, GY785 EPS increased viability and proliferation of the chondrocytes. The hydrogel supplemented with 0.67% GY785 EPS presented an interesting feature for cartilage tissue engineering applications [76].

As described above, with the aim of promoting biological activities, preparations of GY785 derivatives have been undertaken to obtain oversulfated low molecular weight polysaccharides. These derivatives demonstrated a weak anticoagulant activity compared to heparin (10 times less). Two types of derivatives (SDR and DRS) were obtained according to the process used. A process with a first step of sulfation (S) and a subsequent second step of free radical depolymerization (DR) gave the SDR derivatives. A process with a first step of free-radical depolymerization (DR) and a subsequent second step of sulfation (S) resulted in DRS derivatives. Surprisingly, the two types of derivatives did not present the same biological properties. The addition of SDR derivative in the culture medium containing FGF-2 increased the proliferation of mature endothelial cells, whereas the DRS derivative had no effect. In the *in vitro* angiogenesis assay, performed to observe the differentiation of mature endothelial cells into vascular tubes on Matrigel, pre-treatment of cells with SDR derivative increased significantly the formation of vascular tubes, whereas DRS derivatives did not have the same effect. In conclusion SDR GY785 EPS derivative has pro-angiogenic effect contrary to DRS GY785 EPS derivative [77]. However, in the same experimental conditions, SDR GY785 EPS derivative had no effect on proliferation, migration and vascular tube formation on Matrigel from endothelial progenitor cells [72]. In summary, SDR GY785 EPS derivative exhibits pro-angiogenic effect contrary to DRS GY785 EPS derivative, but only in the presence of mature endothelial cells.

In other models of study, it was demonstrated that DRS GY785 EPS stimulated some human mesenchymal cells: dermal fibroblasts, gingival fibroblasts, stromal medullar cells. In two-dimensional cultures, this EPS derivative promoted FGF-2 signaling, and thus cell proliferation. This effect was also observed when cells were associated with extracellular collagenous matrix. Furthermore, DRS GY785 EPS derivatives were able to inhibit some processes involved in tissue breakdown and inflammation such as complement cascade, and induction of MMPs by inflammatory cytokines such as IL-1β and TNF-α [71]. Recently, the effect of this derivative on osteogenesis was investigated because it was previously described that the growth and differentiation of bone cells is controlled by several factors, which can be modulated by heparan sulfates [78]. It was shown that this DRS GY785 EPS derivative inhibited osteoclastogenesis. In addition, the DRS GY785 EPS derivative reduced proliferation and accelerated osteoblastic differentiation, leading to strong inhibition of mineralised nodule formation, which would be in favor of an increase of bone resorption. Taken together, these data show different levels of bone resorption regulation by the DRS GY785 EPS derivative, leading to proresorptive effects [79].

## 5. Conclusions

Marine organisms offer a great diversity of polysaccharides showing interesting biological properties mimicking those described for the mammalian GAGs. Among the different sources of polysaccharides, algal polysaccharides such as fucoidans and especially their LMW derivatives could play an important role in future development of cell therapy and regenerative medicine. Bacterial polysaccharides present also a real potential in cell therapy and tissue engineering with an advantage over the polysaccharides from eukaryotes, since they can be produced totally under controlled conditions in bioreactors. As described for other polysaccharides, some derivatives can be obtained by chemical modifications, to optimize the biological properties and design drugs with improved benefit and low risk for the patient.
